# Complete Genome Sequences of Two Melissococcus plutonius Strains with Different Virulence Profiles, Obtained by PacBio Sequencing

**DOI:** 10.1128/MRA.00038-19

**Published:** 2019-05-23

**Authors:** Kayo Okumura, Daisuke Takamatsu, Masatoshi Okura

**Affiliations:** a Department of Veterinary Medicine, Obihiro University of Agriculture and Veterinary Medicine, Obihiro, Hokkaido, Japan; b Division of Bacterial and Parasitic Disease, National Institute of Animal Health, National Agriculture and Food Research Organization, Tsukuba, Ibaraki, Japan; c The United Graduate School of Veterinary Sciences, Gifu University, Gifu, Japan; Georgia Institute of Technology

## Abstract

Melissococcus plutonius attacks honeybee larvae, causing European foulbrood. Based on their virulence toward larvae, M. plutonius isolates were classified into three types, highly virulent, moderately virulent, and avirulent. We herein performed whole-genome sequencing of *M. plutonius* isolates with different virulence levels to promote an understanding of the pathogenesis of this disease.

## Abstract (precis)

Melissococcus plutonius attacks honeybee larvae, causing European foulbrood. Based on their virulence toward larvae, M. plutonius isolates were classified into three types, highly virulent, moderately virulent, and avirulent.

## ANNOUNCEMENT

The causative agent of European foulbrood, Melissococcus plutonius, infects honeybee larvae, with serious impacts on bee health (1). Based on multilocus sequence typing analysis, M. plutonius isolates were classified into three clonal complexes (CCs), CC3, CC12, and CC13 (2). These CCs exhibited different virulence profiles toward honeybee larvae in experimental infections; CC12 and CC3 strains were extremely and moderately virulent, respectively, whereas the representative CC13 strain was avirulent (3). To clarify the genetic basis of the distinct pathological characteristics of each CC, we performed complete genome sequencing of *M. plutonius* DAT606 and DAT585, which are representative CC3 and CC13 strains, respectively. Previously, we sequenced the genomes of two *M. plutonius* strains, one type strain and one highly virulent strain belonging to CC12 (4, 5). Taken together with the previous genomic data, we have covered all virulence profiles of *M. plutonius*.

*M. plutonius* DAT606 and DAT585 were isolated from diseased European honeybee (Apis mellifera) larvae in Japan (6) and cultured anaerobically on brain heart infusion agar supplemented with KH_2_PO_4_ and starch (KSBHI agar) for 4 days at 35°C. Then, genomic DNA was extracted as described previously, with a slight modification (6); proteinase K treatment was not performed.

Whole-genome sequencing of *M. plutonius* DAT585 and DAT606 was performed on the PacBio (Menlo Park, CA, USA) RS II platform. The library was prepared using single-molecule real-time (SMRT) cell 8Pac V3 and the P6 DNA polymerase binding kit (PacBio), according to the manufacturer’s instructions. Reads were filtered and assembled using SMRT Analysis v2.3 (PacBio) with default settings. The DAT585 genome yielded 100,098 reads encompassing 950,202,716 bp. The mean subread length and *N*_50_ value were 9,492 bp and 13,788 bp, respectively. The DAT606 genome yielded 76,697 reads covering 615,765,788 bp. The mean subread length and *N*_50_ value were 8,028 bp and 12,056 bp, respectively. Subsequently, the filtered reads for the two genomes were assembled *de novo*, producing two circular contigs. As reported previously (5), virulent strains possess another plasmid, pMP19; therefore, for virulent strain DAT606, Sanger sequencing was conducted using conventional primer walking, followed by sequence assembly with Sequencher 5.2 software (Gene Codes, Ann Arbor, MI, USA). Primary coding sequence extraction and initial functional assignment were performed using the automated annotation server RAST*tk* (7). To verify the annotation, the data were inspected and revised manually using the MolecularCloning software v7.07 (In Silico Biology, Kanagawa, Japan). To search phage DNA components in the DAT585 and DAT606 genomes, we used the Web server PHASTER (8).

The chromosomes of both strains contain 60 tRNA genes for all amino acids and four rRNA operons. Additionally, both chromosomes harbor two prophages, one intact and one incomplete. The DAT606 genome contains two plasmids, pMP1 and pMP19, although pMP19 was partially sequenced because of long repeated sequences in a plasmid gene. However, the avirulent strain, DAT585, harbors the pMP1 plasmid only (Table 1).

**TABLE 1:** General features of *Melissococcus plutonius* genomes determined by PacBio sequencing in this study and previous studies

Strain (clonal complex)	Chromosome or plasmid	Size (bp)	No. of coding sequences	No. of pseudogenes	GC content (%)	Accession no. for:		Reference or source
						Complete genome sequence	Raw data	
DAT585 (CC13)	Chromosome	1,890,300	1,481	151	31.4	AP018524	DRA008260	This study
	pMP1	177,500	136	13	29.2	AP018525	DRA008260	This study
DAT606 (CC3)	Chromosome	1,898,117	1,489	148	31.4	AP018526	DRA008261	This study
	pMP1	177,678	136	16	29.2	AP018527	DRA008261	This study
	pMP19	19,989	24	0	30.3	AP018528		This study
DAT561 (CC12)	Chromosome	1,847,807	1,531	18	31.5	AP018492		6
	pMP1	200,057	159	3	29.2	AP018493		6
	pMP19	19,967	28	0	30.3	AP018494		6

### Data availability.

The whole-genome sequences of the chromosome and two plasmids of *M. plutonius* DAT585 and DAT606 were deposited in DDBJ/GenBank under accession numbers AP018524 to AP018528 (Table 1). The raw sequence reads were deposited in the DDBJ Sequence Read Archive (DRA)/NCBI SRA under accession numbers DRA008260 and DRA008261 (Table 1).
